# Anatomical Variations of the Foramen Ovale and Trigeminal Neuralgia

**DOI:** 10.7759/cureus.97572

**Published:** 2025-11-23

**Authors:** Areti Kempa, Natalia Sinou, Nikoleta Sinou, Dimitrios Filippou, Amir Shihada

**Affiliations:** 1 Department of Anatomy, National and Kapodistrian University of Athens School of Medicine, Athens, GRC; 2 Department of Biomedical Sciences, Institute for Research and Education, Athens, GRC; 3 School of Medicine, National and Kapodistrian University of Athens, Athens, GRC

**Keywords:** anatomical variations, fifth cranial nerve, foramen ovale, neuropathy, trigeminal neuralgia

## Abstract

The foramen ovale (FO) is a prominent foramen situated in the middle cranial fossa, allowing passage for a branch of the trigeminal (Vth cranial) nerve, along with the accessory meningeal artery, an emissary vein, and potentially other anatomical features. Initially, it develops as a ring-shaped foramen, but upon complete ossification, its shape varies, with potential differences in size and form as well. This research aims to investigate the diverse anatomical variations of the FO and their implications for the structures traversing it, particularly the third branch of the trigeminal nerve, which may lead to neuropathies such as trigeminal neuralgia. The study utilized databases like PubMed and Scopus, employing the sentence “Anatomical variations of the foramen ovale and trigeminal neuralgia,” along with supplementary retrieval from the bibliographies of relevant articles. In addition to the bibliography retrieved from the bases, a secondary search of articles from the selected bibliographies was also performed. A total of 84 articles were found, of which our study includes 19 articles that meet the selection criteria. The examination revealed numerous possible variations, not only in shape but also in the morphometric properties of the FO. These variations in dimensions and the presence of additional bony formations may influence the anatomical structures passing through it, potentially leading to their entrapment or compression, and resulting in neurological symptoms, specifically trigeminal neuralgia. Consequently, there are documented instances of trigeminal neuralgia linked to vascular compression and the entrapment of the trigeminal nerve. However, further investigation into the various hypotheses surrounding its etiology is essential to elucidate the specific mechanisms underlying the neuropathy, thereby enabling medical specialists to better manage patients with trigeminal neuralgia.

## Introduction and background

The foramen ovale (FO) is found in the floor of the middle cranial fossa, positioned in the posterior region of the greater wing of the sphenoid bone, anteromedial to the foramen spinosum (FS) and posterolateral to the foramen rotundum (FR) [1,2]. Several anatomical structures pass through it, including the mandibular branch of the trigeminal nerve (V3), the accessory middle meningeal artery, emissary veins, the anterior trunk of the middle meningeal sinus, and sometimes the lesser petrosal nerve [2]. From an embryological perspective, the mandibular nerve traverses the FO during intrauterine development, surrounded by membranous bone. The initial ossification center appears by the eighth week, with the first formation of a ring-shaped FO observed by the end of the seventh month of fetal life [3]. As development progresses, the FO may undergo differentiation, leading to anatomical variations, morphometric changes, and the potential emergence of bony ligaments and spurs. Although these variations are often benign, if they impinge upon or entrap anatomical structures traversing the FO, they could provoke trigeminal neuralgia (TN) due to the involvement of the mandibular nerve (V3) located within the FO. Additionally, the presence of a tumor near this structure may compress the trigeminal nerve, resulting in pain and other symptoms associated with TN.

Trigeminal neuralgia is a chronic pain condition primarily affecting the facial region, resulting from compression of the trigeminal nerve, also referred to as the fifth cranial nerve. The prevalence of TN in the general global population is estimated to be about 3-5 cases per 100,000 individuals, while the annual incidence ranges from 4.5 to 28.9 cases per 100,000, increasing with age [4]. The symptoms associated with TN include severe facial pain and brief pain episodes that can occur not just during eating and jaw movement but throughout the day. This significantly impacts patients' lives, making daily activities like eating and speaking challenging, thereby reducing their overall quality of life.

The trigeminal nerve is one of the most intricate cranial nerves, featuring both a sensory root and a smaller motor root that handle facial sensation and basic motor functions like chewing, respectively. The trigeminal ganglion is situated within Meckel’s cave, which permits the three major branches of the nerve to emerge. These key branches are the ophthalmic (V1), maxillary (V2), and mandibular (V3) nerves, which exit the inner cranial fossa via the superior orbital fissure (FOS), foramen rotundum (FR), and foramen ovale (FO), respectively [5].

Several case reports have substantiated these potential causes, and other hypotheses regarding the increased incidence of TN on the right side of the cranial nerve, as well as possible peripheral origins, have also been proposed. Therefore, understanding the anatomical specifics and variations of the FO is of paramount importance, given that the etiology of TN can differ widely, with many causes still remaining unidentified. The aim of this study is to discuss the various anatomical variations, the morphology and morphometry of the FO, as well as their potential correlation with the onset of TN, supported by case reports and theoretical hypotheses regarding the possible mechanisms that may lead to its development.

## Review

Materials and methods

The current investigation was structured as a systematic review, relying on a compilation of data sourced from specialized medical websites, particularly PubMed and Scopus. The data were gathered using the searching phrase “Anatomical variations of the foramen ovale and trigeminal neuralgia,” which relates to the anatomical features of the FO and potential variations contributing to TN.

**Figure 1:**
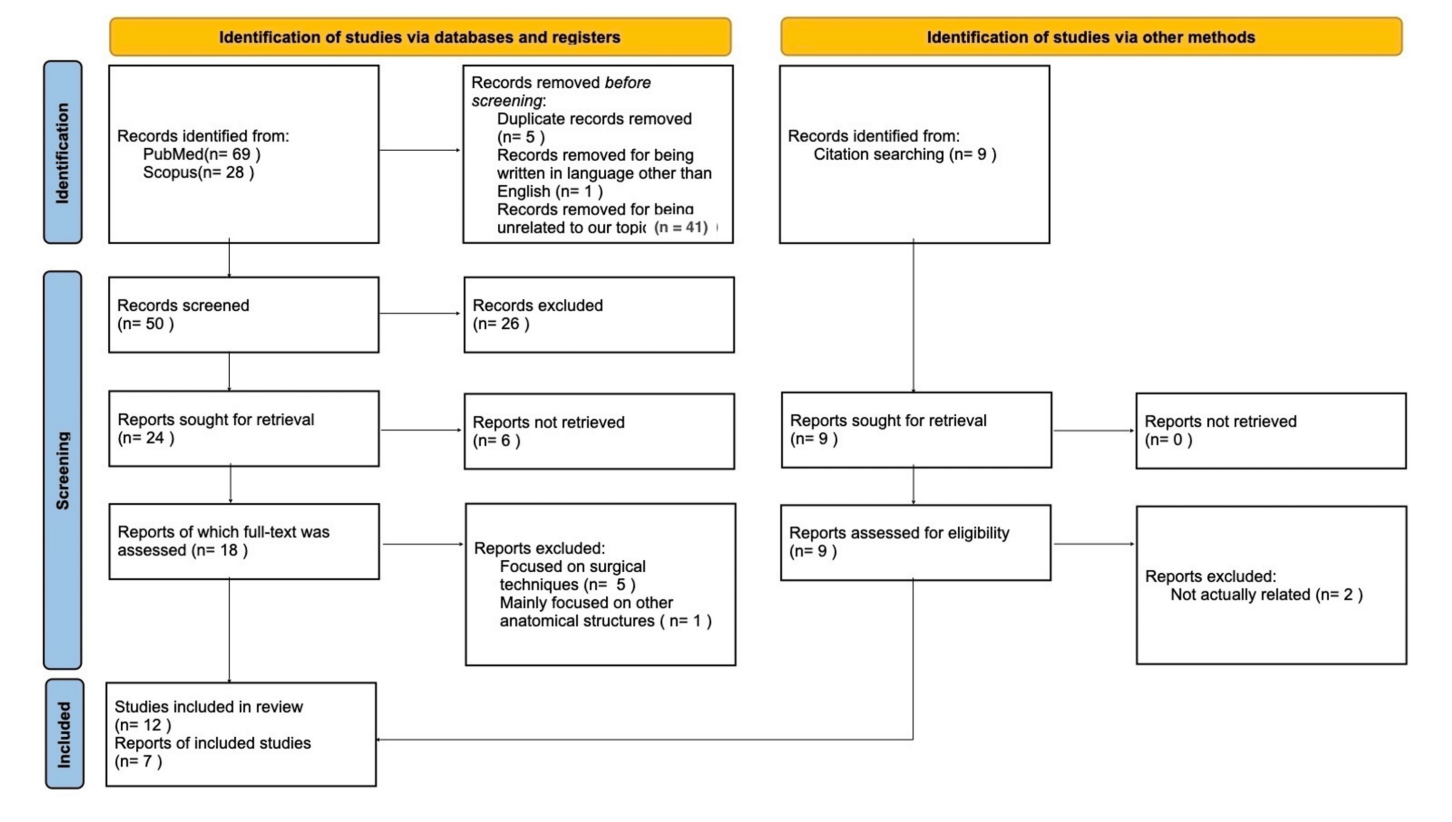
PRISMA 2020 flow diagram for new systematic reviews which included searches of databases, registers and other sources. PRISMA: Preferred Reporting Items for Systematic Reviews and Meta-Analyses

Specifically, the study adhered to the Preferred Reporting Items for Systematic Reviews and Meta-Analyses (PRISMA) 2020 flow diagram guidelines for systematic reviews, focusing exclusively on database searches (Figure 1). In total, 69 articles were found on PubMed and 28 on Scopus based on the specified search algorithm, although 41 of these were deemed irrelevant to our subject matter. From the remaining 56 articles, one was excluded for being in a non-English language, and five articles from Scopus were duplicates already identified in PubMed, with no additional duplicates present. Consequently, 50 articles remained. A review of the abstracts led to the identification of 24 articles pertinent to our research, while the other 26 mainly focused either on the anatomy of the FO or on TN, but not on the correlation of these two. Ultimately, after a thorough examination of the full texts, only 12 articles met the criteria for inclusion and discussed the connection between the FO's anatomy and TN. An additional seven articles were incorporated following a secondary search of the references from the initially approved 12. In total, 19 articles were utilized in this study.

Results

From the 19 articles in total, which were included in this study, three presented clinical cases explaining the etiology of TN in three totally different patient cases. There were also articles that present possible pathological mechanisms regarding the causes of trigeminal neuralgia. From the remaining 14 articles, in articles [1-3,6-11], they studied the anatomical variations of FO on dried human skulls. After comparing their results, all articles that observed the shape of the FO [1-3,6,7] found it to be most commonly ‘’oval shaped’’ (Table 1). Kodialbail et al. found the width of the FO was significantly smaller on the right side in women's adult skulls, but Kaur et al. found the anteroposterior distance on the right-sided FOs had a larger diameter, while Natsis et al. found no significant differences in the morphometric measurements in the fifty skulls that were measured in their study [1,3,7]. The existence of bony outgrowths such as the pterygoalar and pterygospinous bar, due to the complete or incomplete ossification of the corresponding ligaments, is mentioned by Kodialbail et al., believed to be age-related, fact supported by Natsis et al. too but denied by Shaw, where they mostly correlate the formation of such outgrowths with genetic factors, after having observed their existence, in cases of young people, even children [1,7,10]. Many specimens with either a complete or incomplete pterygoalar and pterygospinous bar were found in a few articles [3,6-12], occurring on the right or left side of the skull, and in some cases, even bilaterally. There are also three cases mentioned by Khairnar and Bhusari that presented with a doubled FO [6].

**Table 1:** Variations in the shape of the FO. * Only percentages were available. FO: foramen ovale.

Article	Method used	Number of FOs	Oval		Almond		Round		D-shaped		Slit-like		Irregular		Triagular		Pear shaped		Elongated		Kidney	
-	-	-	Right	Left	Right	Left	Right	Left	Right	Left	Right	Left	Right	Left	Right	Left	Right	Left	Right	Left	Right	Left
Kodialbail et al. [1]	Dries skull	100	56		26		4		-		14		-		-		-		-		-	
Kaur et al. [3]	Dries skull	200	68	72	20	18	4	2	6	6	1	1	-	-	1	-	-	1	-	-	-	-
Khairnar and Bhusari [6]	Dries skull	200	78	75	9	12	6	8	-	-	7	5	-	-	-	-	-	-	-	-	-	-
Kastamoni et al. [4]	CT scan	337	273		51		7		-		-		-		-		-		-		6	
Natsis K et al. [7]*	Dries skull	232	62.1%	49.1%	24.1%	14.7%	6.9%	8.6%	-	-	-	-	19.8%	14.7%	-	-	-	-	-	-	-	-

In the last four articles included in this study, they used a different method to measure the morphometric characteristics and observe any anatomical variations of the FO [4,5,13,14]. More precisely, they used a CT scan to collect all the information they needed from their specimens. The dominant shape for the FO was again found to be ‘’oval’’ (Table 1). Although Liu et al. support that there is no correlation between the dimensions of the FO and gender, Kastamoni et al. observed narrower width, length, and area of the FO in female subjects [4,13]. Then, a significant difference in the dimensions of the FO between the painful and pain-free sides of patients with TN is observed in all articles using the CT method, while a relation between advancing age and the presence of TN is also suggested by Aksoy et al. [5]. Lastly, in Kastamoni et al., both cases with bony spurs and growths are mentioned, alongside a single case with a doubled FO [4].

Discussion

The FO exhibits a range of anatomical variations, encompassing not only its morphometric features and shapes but also the presence of bony outgrowths. Studies conducted on dry human skulls using both Vernier and digital calipers revealed diverse FO shapes. The "oval" configuration was predominant, followed by "almond," "round," "slit-like," and in some instances, "triangular," "D-shaped," and "pear-shaped" were also identified [1,3,6]. Of the numerous diameters examined in various studies, one found that the anteroposterior distance was statistically significantly shorter on the right side compared to the left side of the infratemporal fossa with a p-value of p=0.0194 [3]. In another investigation, statistically significant discrepancies were noted between the paired left and right major axes (p=0.000003), aspect ratio (p=0.018942), area (p=0.000125), perimeter (p=0.001240), and roundness (p=0.012339) of the FO, while circularity and solidity showed no significant differences [2]. Additionally, a study involving 116 Greek adult skulls found no correlation between FO shape and gender; however, a significant difference was observed in the surface area of the FO, revealing that female subjects had smaller measurements on both right (p=0.012) and left sides (p=0.04). Their measurements indicated a significantly reduced width on the right side as well (p=0.02) [7].

Moreover, multiple studies document the presence of bony outgrowths, including tubercles, spurs, pterygospinous bars, or pterygoalar bars; these can either develop entirely or partially and may exist unilaterally or bilaterally [1,3,6-12]. Some research indicates a predisposition for the incomplete pterygoalar or pterygospinous bars to be located on the left side of the infratemporal fossa, with the pterygospinous bar typically running medial or inferior to the FO, while the pterygoalar bar's course is lateral, medial, or inferior [8]; however, another study found no side preference in their analysis of 145 human dry skulls [9]. The formation of these structures is believed to result from developmental processes associated with the ossification of the pterygoalar or pterygospinous ligament occurring alongside the cranial cavity development and the complete morphology of the FO. The occurrence of a complete pterygoalar bar tends to correlate with age, particularly becoming more common in individuals over 40, with a statistically significant difference (p<0.001) [9]. Contrastingly, in two other studies was found no age correlation was found linked to fully ossified pterygoalar or pterygospinous bars, as they noted complete formations in younger individuals, including children, implying possible genetic influences [10,12]. Generally, the presence of a pterygospinous or pterygoalar bar-regardless of its completeness-can lead to entrapment or compression of anatomical structures traversing the FO, such as the lingual nerve (a branch of the trigeminal nerve, V3), various V3 branches, or even the main V3 trunk, contingent on how the bony outgrowth forms, potentially dividing the foramen into smaller segments [11,12]. Additionally, such bony bridges might compress the accessory meningeal artery, obstructing blood supply to the trigeminal ganglion [8]. Beyond unique anatomical structures, instances of a duplicated FO or connections between the FO and FS have been recorded in different studies, impacting the pathways of nerves and blood vessels that ordinarily pass through these foramina, which may lead to neurological symptoms, such as TN [6].

Furthermore, several studies utilized CT imaging techniques to examine and quantify the FO dimensions and other anatomical variations. They consistently found that the predominant shape of the FO was "oval," followed by variations such as "almond," "round," "slit-like," "D-shaped," and "irregular" [4]. Measurements of length, width, and area indicated no statistical difference between the left and right sides in patients with trigeminal neuralgia [4,5,13,14]. However, a significant statistical difference was evident when comparing the width and area of the FO between the painful side in trigeminal neuralgia patients and asymptomatic individuals, although no significant differences in length were observed [4,5,13,14] The observed narrowing of the FO on the painful side of TN patients suggests a correlation between the reduced size of the foramen and potential compression of the branches of the fifth cranial nerve, leading to trigeminal neuralgia. This outlines how the nerve can become entrapped, leading to demyelination of its axons while also implicating other anatomical structures traversing the foramen, like the venous connection between the pterygoalar venous plexus and the cavernous sinus, accessory meningeal artery, and emissary vein, potentially affecting blood flow to the trigeminal ganglion and causing symptoms of Vth cranial nerve neuralgia [13]. Moreover, FO dimensions-length, width, and area-were found to be smaller in female subjects compared to male subjects, supporting the previously observed higher incidence of TN in female subjects, estimated to be twice as frequent [4]. The existence of a second FO has been mentioned in some studies, although such cases are rare [4].

Upon comparing measurement methods used to observe anatomical variations of the FO, it becomes evident that conclusions such as the prevalent "oval" shape, the reduced width and area in female subjects, and generally smaller dimensions in individuals with trigeminal neuralgia align closely. Nevertheless, specific anatomical measurements vary between studies (Table 2), attributed to differing measurement tools, techniques, and the limited sample sizes due to the rarity of TN cases and variation in study criteria and parameters [14].

**Table 2:** Measurements of the foramen ovale and comparison of them, collected from the included studies. FO: foramen ovale, TN: trigeminal neuralgia, CT: computed tomography.

FO measurements	Year	Sample size and type	Asymptomatic individuals				TN patients			
			length (mm)		width (mm)		length (mm)		width (mm)	
-	-	-	right	left	right	left	painful side	pain-free side	painful side	pain-free side
Kastamoni et al. [4]	2021	158 CT scans from asympomatic individuals.	6.05+-1.01	5.86+-0.92	3.35+-0.83	3.37+-0.75	-			
		19 samples from TN patients (21 samples on painful side and 17 on pain-free side)	-				5.38+-1.54	5.53+-1.38	2.88+-0.83	2.83+-0.68
											Aksoy et al. [5]	2021	40 CT scans from healthy individuals	7.9+-1.03	7.8+-0.76	5.8+-0.77	5.8+-0.65	-			
40 CT scans from TN patients	-				6.3+-1.14	6.6+-0.79	4.7+-1.20	4.8+-0.66				
Kodialbail et al. [1]	2024	50 dry human skulls	7.97+- 1.07	7.78+-1.15	4.62+-1.18	4.55+-0.07	-			
Zdilla et al. [2]	2016	91 dry human skulls	6.62+-1.12	5.99+-1.08	3.13+-0.66	3.02+-0.63	-			
Kaur et al. [3]	2022	100 dry human skulls	8.16+-1.560	7.68+-1.253	4.97+-1.164	4.74+-1.206	-			
Natsis et al. [7]	2015	116 dry human skulls	7.2+-1.2	7.0+-1.1	3.9+-0.7	4.1+-0.8	-			
Liu et al. [13]	2016	30 CT scans from healthy individuals	7.56+-1.55	7.69+-1.44	3.76+-0.80	3.89+-0.72	-			
		21 CT scans from TN patients	-				7.59+-1.35	7.68+-1.51	3.82+-0.65	3.99+-0.87
Li et al. [14]	2022	46 CT scans from healthy individuals	5.79	5.27	2.77	2.64	-			
		79 CT scans from classical TN patients	-				5.36	5.22	2.45	2.45
		30 CT scans from idiopathic TN patients	-				5.38	5.10	2.55	2.49

In a case report, a 53-year-old woman presented with classic right-sided TN affecting the second and third branches of the trigeminal nerve. Following examination, microvascular decompression surgery was recommended. The superior cerebellar artery was found to be in a normal position; however, a large petrosal vein was compressing the inferolateral aspect of the trigeminal sensory root. After this vein was excised, a small arteriole was noted to be compressing some nerve fibers on the deep surface. This abnormal area was subsequently removed, leading to the successful completion of the procedure. An electron microscopy analysis revealed demyelinated nerve fibers, with no inflammation observed. The combination of the previously compressed and demyelinated nerve fibers, along with ephaptic transmission, was identified as the cause of trigeminal neuralgia. Upon removal of the compressive blood vessel, the patient's symptoms were alleviated as well [15].

In another instance involving a 49-year-old woman, she presented with left facial numbness persisting for four years, along with recent difficulties in chewing, and new numbness in her right lip just prior to admission. Examination revealed an area of sensory impairment in the third branch of the trigeminal nerve and mild swelling of the left trigeminal nerve within the cavernous sinus, but no other neurological symptoms were detected. During surgery, an amorphous mass resembling a tumor was identified behind the trigeminal nerve ganglion after the dura was incised. Postoperative biopsy confirmed infiltration of the trigeminal nerve and the left gasserian ganglion by amorphous masses, identified as an amyloidoma associated with symptomatic involvement of the contralateral trigeminal nerve. Thus, in this case, the TN was attributed to a tumor rather than vascular compression or other previously mentioned causes [16].

The third interesting case concerns an 83-year-old male. Upon dissection, the right mandibular nerve was exposed, while the lingual nerve exhibited an atypical course and entrapment due to an ossified pterygospinous ligament, which typically appears as a bifurcated foramen ovale in axial skull projection. The ossified pterygospinous bar, combined with the atypical pathway of the lingual nerve, may have caused numbness in the area innervated by the nerve, as well as pain during speech [17].

Possible mechanisms behind TN related to the anatomy of the FO reveal that this severe condition, with uncertain causes, has several hypotheses about the mechanisms leading to neuralgia of the Vth cranial nerve. One theory suggests that vascular compression leads to inflammation of the trigeminal nerve, resulting in entrapment as it traverses the FO. Notably, it has been observed that TN is more prevalent on the right side, potentially due to the FO being narrower on that side [18]. Consequently, enlargement of the mandibular nerve could lead to nerve entrapment in the FO, contributing to neuralgia, particularly on the right, alongside other underlying factors. This theory is supported by instances of coexistence of multiple sclerosis and TN, in which cases a right-sided preference remains, unlike when TN is absent, as well as with tumors and cysts [18]. Moreover, entrapment within the FO may result in demyelination of certain nerve fibers, causing them to fire upon contact with intact fibers, manifesting as paroxysmal pain and perpetuating the short-circuiting phenomenon. This could lead to further demyelination, creating additional trigger points and increasing the intensity of the paroxysmal pain [19].

In this systematic review, although a comprehensive search strategy was followed, some relevant studies may not have been included cause of the use of language other than English. Also, the heterogeneity of the studies may have limited the ability of the meta-analysis. Lastly, the variety of tools and precision used in each study may have impaired the specific comparison of some data. Despite these limitations, this systematic review provides a meaningful synthesis of the current literature.

## Conclusions

In conclusion, the foramen ovale (FO) is situated in the middle cranial fossa, located near the foramen rotundum and foramen spinosum, specifically posteriolateral and anteriomedial to them, respectively. The formation of the foramen ovale occurs during fetal development, typically resulting in a ring-shaped FO after the seventh month, with continued development possibly leading to anatomical changes in shape, size, and the formation of related structures. Understanding the precise morphometric features and dimensions of the foramen ovale, along with potential bony outgrowths or abnormalities, holds significant importance, as these may contribute to various neurological symptoms, notably trigeminal neuralgia due to entrapment or compression of nearby anatomical structures such as the mandibular nerve and accessory meningeal artery. The etiology of this pathology remains largely unknown, but numerous hypotheses have emerged and continue to evolve. Additional case reports of trigeminal neuralgia patients can provide deeper insights into the mechanisms behind this condition and raise awareness. Extensive research is warranted, given many mechanisms are still unclear and several remain undiscovered. Improving our understanding of the pathology of trigeminal neuralgia could positively influence management and treatment options for affected patients. This information is particularly valuable for neurologists and neurosurgeons involved in treating individuals with trigeminal neuralgia.
